# Inhibition of Casein Kinase 1*δ* as a Novel Therapeutic Strategy for Amyotrophic Lateral Sclerosis: A Theoretical Study

**DOI:** 10.3390/ijms262010188

**Published:** 2025-10-20

**Authors:** Albert Gabriel Turpo-Peqqueña, Renato Javier Valencia-Arce, Fabio Leonardo Del-Carpio-Carrazco, David Jonatan Quispe-Ppacco, Pierina Fernanda Carbajal-Llerena, Harlly Romed Loza-Chipa, Antonella Sofia Vásquez-Macedo, Badhin Gómez

**Affiliations:** 1 Centro de Investigación en Ingeniería Molecular–CIIM, Universidad Católica de Santa María, Urb. San José s/n, Umacollo, Arequipa 04013, Peru; albert.turpo@estudiante.ucsm.edu.pe (A.G.T.-P.); 71505948@estudiante.ucsm.edu.pe (R.J.V.-A.); 70666250@ucsm.edu.pe (F.L.D.-C.-C.); david.quispe@estudiante.ucsm.edu.pe (D.J.Q.-P.); pierina.carbajal@estudiante.ucsm.edu.pe (P.F.C.-L.); harlly.loza@estudiante.ucsm.edu.pe (H.R.L.-C.); antonella.vasquez@estudiante.ucsm.edu.pe (A.S.V.-M.); 2 Facultad de Medicina Humana, Centro de Investigación y Estudios Médicos (CIEM), Universidad Católica de Santa María, Urb. San José s/n, Arequipa 04013, Peru; 3 Facultad de Biología, Universidad Nacional de San Agustín, Av. Alcides Carrión s/n, Arequipa 04000, Peru; 4 Facultad de Ciencias Farmacéuticas, Bioquímicas y Biotecnológicas, Universidad Católica de Santa María, Urb. San José s/n, Umacollo, Arequipa 04013, Peru

**Keywords:** amyotrophic lateral sclerosis (ALS), CK1δ, molecular docking, molecular dynamics, MMPBSA, virtual screening, neurodegeneration

## Abstract

Amyotrophic Lateral Sclerosis is a progressive neurodegenerative disease characterized by the degeneration of motor neurons and the pathological accumulation of phosphorylated TDP-43. Casein kinase one delta (CK1δ) has been identified as a key regulator of this aberrant phosphorylation, making it a promising therapeutic target. In this theoretical study, 26 structurally diverse compounds were evaluated against CK1δ using molecular docking, molecular dynamics simulations, and binding free energy calculations. Among them, BZH exhibited the most stable interaction with CK1δ (−46.53±1.94 kcal/mol). An inverse correlation was observed between theoretical affinity and experimental IC_50_ values, supporting the predictive validity of the computational approach. Pharmacokinetic analysis indicated that IMF and BIP show good oral absorption and the ability to cross the blood–brain barrier. At the same time, the toxicological profile classified all compounds in toxicity Class IV (moderate risk). Additionally, dynamic migration toward an alternative pocket was observed during simulation, highlighting the importance of considering protein flexibility in drug design. This study proposes BZH, IMF, and BIP as promising CK1δ inhibitors for future experimental validation in the treatment of ALS.

## 1. Introduction

Neurodegenerative diseases (NDs) are the leading cause of physical and cognitive disability worldwide, affecting around 15% of the global population [1]. Among the NDs, Amyotrophic Lateral Sclerosis (ALS) is rare, heterogeneous, progressive, and fatal, affecting the central nervous system [2]. Its hallmark feature is the simultaneous degeneration of upper motor neurons, located in the motor cortex, and lower motor neurons, which connect the spinal cord to the muscles. This neuronal damage leads to both motor and extra-motor clinical signs such as muscle weakness, atrophy, cramps, fasciculations, spasticity, clumsiness, and hyperreflexia. Over time, these symptoms lead to generalized paralysis that eventually results in the death of the patient, commonly due to respiratory failure [3,4,5]. Approximately 90% of ALS cases are sporadic, with no apparent heritability, while 10% are familial or genetic in origin [6,7]. ALS incidence peaks between the ages of 60 and 79, with a higher prevalence in males [8], and an estimated incidence rate of 1.68 per 100,000 people per year, depending on the region [9]. Despite scientific advances in this disease, the prognosis remains poor, with a median survival of 3 to 5 years after diagnosis [4]. To date, there is no definitive cure, and there are only a few therapies that slow disease progression. One such therapy is Riluzole, the only FDA-approved drug, which has been shown to extend survival in some cases by up to 3 months [10,11]. Therefore, there is an urgent need to develop innovative therapeutic approaches that improve the quality of life for these patients. ALS lacks a single etiology and is caused by the interaction of diverse molecular pathways [6,12].

One of the most critical findings in recent years has been the pathological involvement of the DNA-binding protein TAR 43 (TDP-43) [13,14,15], encoded by the *TARDBP* gene, which regulates RNA processes in the nucleus, such as transcriptional regulation, alternative splicing, and mRNA stabilization. However, under pathological conditions, TDP-43 undergoes cytoplasmic mislocalization, accompanied by hyperphosphorylation and other modifications that promote its accumulation and the formation of toxic cytoplasmic inclusions [16]. The cytoplasmic mislocalization of TDP-43 is a hallmark and key toxic event in ALS, although the mechanisms regulating it are not yet fully understood [17]. Among these regulators, casein kinase one delta (CK1δ), a serine/threonine enzyme involved in multiple cellular processes, has been identified as one of the main contributors to the aberrant phosphorylation of TDP-43 [18]. In the report by Choksi [19], it was demonstrated that phosphorylation of TDP-43 by CK1ε promoted oligomerization and increased in vivo toxicity. Similarly, in the study by Hicks [20], endoplasmic reticulum stress induced the cytoplasmic accumulation of phosphorylated TDP-43 in a CK1-dependent manner in motor neuron-like cells. Furthermore, Liachko [21] showed in *C. elegans* that phosphorylation of TDP-43 by kinases such as CK1 significantly increased its neurotoxicity, as evidenced by motor dysfunction and motor neuron degeneration. Finally, Martínez-González [22] reported that inhibition of CK1δ reduced the aberrant phosphorylation of TDP-43, attenuated its toxicity, and preserved motor neurons in both cellular and murine models of ALS. Due to the critical role of CK1δ in the pathological phosphorylation of TDP-43, its inhibition presents a promising therapeutic strategy in ALS.

However, current CK1δ inhibitors still present experimental limitations regarding their specificity. Therefore, molecular modeling, molecular docking, and molecular dynamics simulations could provide a detailed understanding of protein behavior at the molecular level and help develop greater specificity toward potential inhibitors [23]. The objective of this study is to identify potential inhibitors targeting CK1δ through molecular dynamics simulations and to provide theoretical insights for the development of optimized experimental studies in ALS.

## 2. Results

The structure of CK1δ, obtained from the PDB database (ID: 6RCG) [24], contained multiple chains and several crystallographic water molecules. We used UCSF Chimera v1.18 software [25] to remove all extraneous molecules, including water and potential ions. Missing residues in the CK1δ sequence were identified and reconstructed by homology modeling with the MODELLER tool [26] integrated into Chimera. The processed structure of CK1δ, composed of 296 residues, was solvated in a cubic box of 8.3436 nm per side using the TIP3P water model. The addition of Cl^−^ atoms neutralized the net charge of the system, and additional Na^+^ and Cl^−^ ions were incorporated to reach a physiological ionic concentration of 0.15 M. In total, we added 17,340 water molecules to the system.

**Figure 1:**
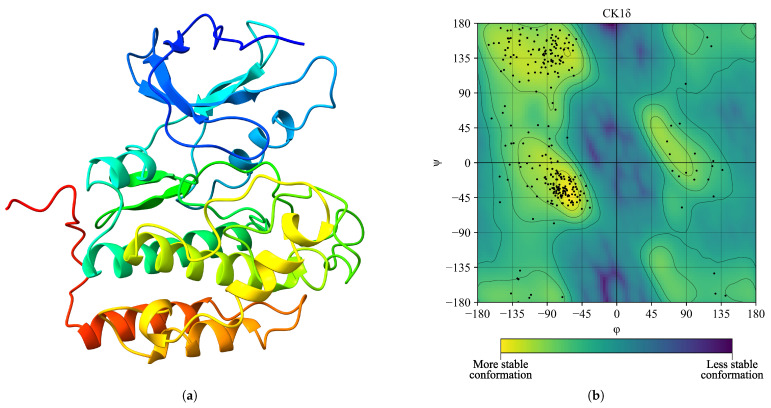
Stabilized structure of CK1δ (ID: 6RCG) and residue stability analysis after 500 ns of simulation. (**a**) Structure of CK1δ. (**b**) Ramachandran diagram, the yellow color shows a more stable conformation and purple color shows a less stable conformation.

Furthermore, we minimized the forces on the system using the steepest descent algorithm down to a tolerance of 1.0 kJ/mol. Next, we followed with thermal (309.65 K) and pressure (1 bar) equilibration steps, both lasting 10 ns. A 500 ns molecular dynamics simulation with one fs steps was then run (see Figure 1a). We performed Ramachandran diagram analysis to assess the structural conformation of the CK1δ protein. Of the 261 non-glycine/proline residues analyzed, 221 residues (84.7%) were located in the most favored regions, 34 (13.0%) in additionally allowed regions, and 5 (1.9%) in generously allowed regions, representing 99.6% of the residues. We located only one residue (0.4%) in the disallowed regions. This evaluation excluded glycine and proline residues, as well as terminal residues, from the analysis (see Figure 1b).

**Figure 2:**
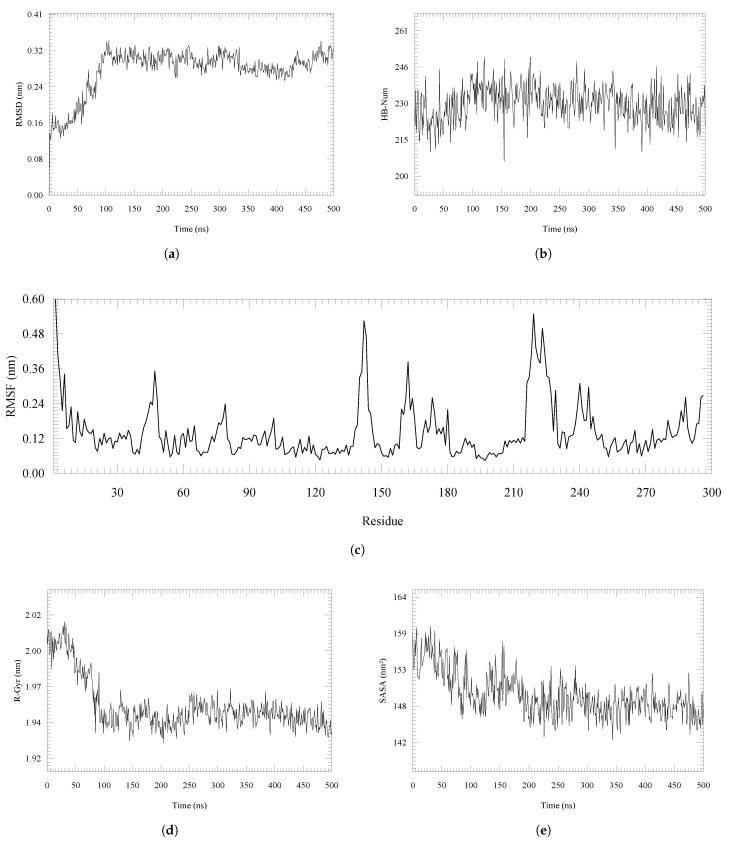
Structural properties of CK1δ during a 500 ns molecular dynamics simulation. (**a**) Root mean square deviation (RMSD). (**b**) Number of hydrogen bonds. (**c**) Root mean square fluctuation per residue (RMSF). (**d**) Radius of gyration (R-Gyr). (**e**) Solvent-accessible surface area (SASA).

By analyzing the root mean square deviation (RMSD) of the distances (see Figure 2a), we observe that the system reaches a state of structural equilibrium after 100 ns, with average fluctuations of around 0.3 nm until the end of the molecular dynamics simulation, indicating overall stability of the protein conformation. In Figure 2b, we present the variation in hydrogen bonds in the protein structure during the molecular dynamics simulation trajectory. We obtained an almost constant average of 230 bonds throughout the simulation, reflecting stable interactions.

In the root mean square deviation (RMSD) of the residue (see Figure 2c), we have identified peaks of higher mobility between residues near positions 144 to 150 and between 220 and 255, probably in flexible regions. Likewise, we observe concerning the radius of gyration (see Figure 2d), an initial compaction process that stabilizes after 100 ns, with constant values around 1.94 nm. Finally, the solvent accessible surface area (SASA) (see Figure 2e) shows a gradual decrease during the first 150 ns, followed by a stabilization phase at 200 nm, reflecting the clustering of the observed structure.

**Figure 3:**
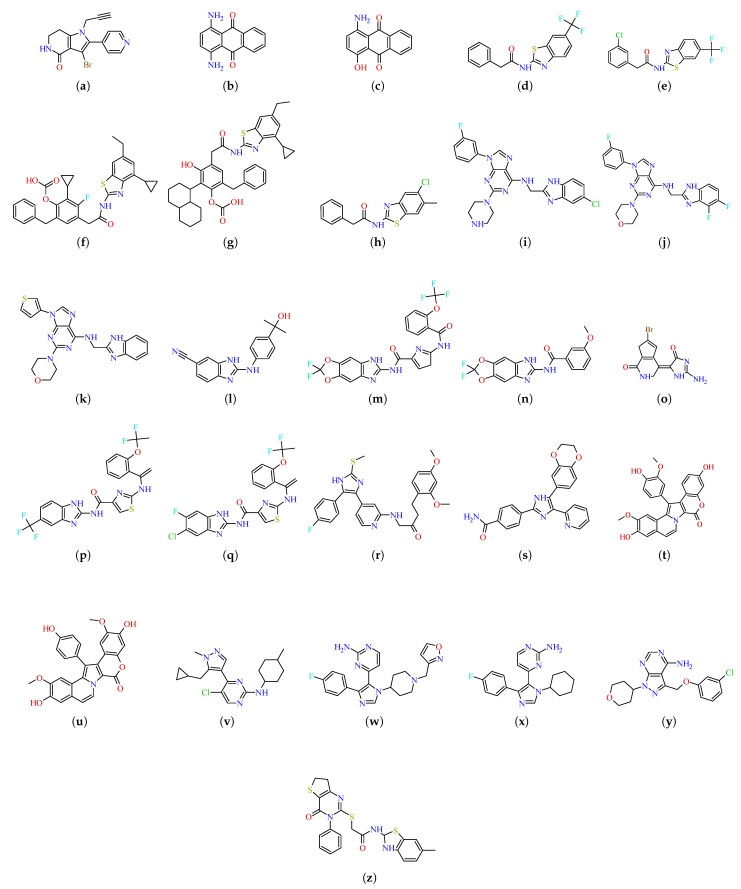
Two-dimensional structures of all compounds classified by chemical family: Amine, Benzimidazole, Benzothiazole, Imidazole, Lamellarin, Pyrimidine, and Triazatin. (**a**) AMI. (**b**) AMQ. (**c**) AHQ. (**d**) BZF. (**e**) BZT. (**f**) BZC. (**g**) BZH. (**h**) BZM. (**i**) BIP. (**j**) BIM. (**k**) BIT. (**l**) BIC. (**m**) DXB. (**n**) DXM. (**o**) HYA. (**p**) IMT. (**q**) IMF. (**r**) IMB. (**s**) IMD. (**t**) LAM. (**u**) LAX. (**v**) PMP. (**w**) PYP. (**x**) PYI. (**y**) PYR. (**z**) TTA.

In Figure 3, we present the two-dimensional (2D) structures that we identified in databases with potential inhibitory activity against CK1δ. We have found potential ligands from the families of amines, benzimidazoles, benzothiazoles, imidazoles, lamellarines, pyrimidines, and triazatines. Among all the structures, we identified two as hydrochlorides (AMI and PYR).

Pharmacologically relevant cavities of the CK1δ protein were analyzed using the CavityPlus server [27] to identify potential inhibitor binding regions. Table 1 presents the results for the nine cavities with the highest binding potential. The highest scoring cavity obtained a value of 1058, while the lowest scored reached −1274, in arbitrary pharmacological units. In general, the higher the DrugScore value, the higher the probability that a molecule can interact favorably with that region of the protein. Conversely, negative values suggest a low probability of interaction. It is important to emphasize that we based this analysis on a static protein structure; under physiological conditions, flexibility and structural motion can influence the binding affinity of inhibitors.

**Figure 4:**
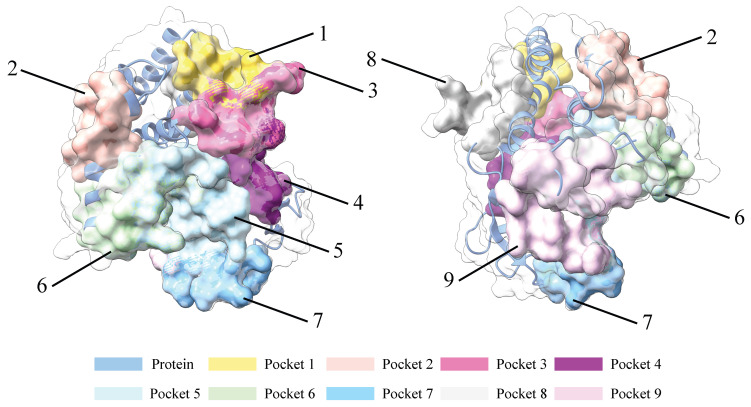
Binding pockets identified in the CK1δ protein.

Figure 4 shows the binding pockets identified in the CK1δ protein using the CavityPlus server. Each pocket is marked with a specific color and numbered according to the data presented in Table 1. The colors are only referential; they have no meaning except to identify them.

We used UNI-DOCK [28] to dock the inhibitors to the stabilized CK1δ protein. For each ligand, we performed 2500 simulation events, resulting in a total of 75,000 dockings. In each case, we selected the complex with the lowest predicted affinity energy or the one with the greatest absolute energy (see Table 2).

It is important to emphasize that the energy values presented are for reference only; we employ force fields that use geometric parameters, such as those derived from Van der Waals volumes. When analyzing the binding energy results for the complexes with CK1δ, six compounds with the highest affinities were identified: DXB with a binding energy of −8.14 kcal/mol, followed by BIM (−7.70 kcal/mol), BZH (−7.66 kcal/mol), BIP (−7.32 kcal/mol), IMT (−7.21 kcal/mol), and IMF (−7.17 kcal/mol). In contrast, the compound with the lowest affinity was AMQ, with a binding energy of −5.27 kcal/mol. The docking energies obtained in Table 2 are only referential, since they are based on probabilistic models and simplified force fields. To obtain more accurate interaction data, we performed 100 ns molecular dynamics simulations under constant physiological conditions. The last 10 ns was analyzed using the MMPBSA method, evaluating the interaction free energy and its energy components.

Table 2 shows the interaction free energies between CK1δ and the different inhibitors, presenting a binding free energy scale of BZH > IMF > IMB > TTA > BIP. The value for BZH was −46.53 ± 1.94 kcal/mol, while for BIP it was −31.44 ± 0.99 kcal/mol. When analyzing the binding free energies by Van der Waals interactions, we observed the following order: BZH > IMF > TTA > IMB > BIP. The value for BZH was −52.70 ± 1.93 kcal/mol, while for BIP it was −35.48 ± 0.94 kcal/mol. In the case of electrostatic interactions, the order was IMB > BIP > BZH > TTA > IMF. The highest value was for IMB with −6.30 ± 0.62 kcal/mol, and the lowest for IMF with −2.27 ± 1.28 kcal/mol. Regarding the polar solvation energy, we observed the following order: BZH > IMB > TTA > BIP > IMF. The highest value was for BZH with 16.37 ± 0.05 kcal/mol, while the lowest value was for IMF with 10.76 ± 0.45 kcal/mol. When examining the solvent accessible surface area (SASA), we found the following order: BZH > IMF > IMB > TTA > BIP. BZH showed the most favorable contribution with −6.07 ± 0.09 kcal/mol, while BIP showed the least favorable with −3.79 ± 0.01 kcal/mol. Finally, regarding the contribution of solvent-accessible volume (SAV), the order was IMB > BZH > TTA > BIP > IMF. The highest value was for IMB, with 10.56 ± 0.26 kcal/mol, and the lowest for IMF, with 6.25 ± 0.45 kcal/mol. These results suggest that the compound BZH exhibits potential inhibitory activity against CK1δ.

Among the twenty-six structurally diverse compounds analyzed through molecular docking using UNI-DOCK [28], DXB initially showed the highest predicted binding affinity (−8.14 kcal/mol), followed by BIM, BZH, and BIP. However, this ranking changed significantly when we performed molecular dynamics simulations and MMPBSA binding free energy calculations. BZH emerged as the most stable and promising inhibitor, with the most favorable free energy (−46.53 ± 1.94 kcal/mol), while DXB showed a less favorable interaction profile (−20.16 ± 3.45 kcal/mol). These findings underscore the limitations of relying solely on static docking scores, which do not account for conformational dynamics or solvent effects. The MMPBSA decomposition further highlighted BZH’s strong Van der Waals interactions and favorable SASA contributions. Altogether, this reinforces the need to integrate dynamic methods, such as MMPBSA, to better reflect the real biophysical behavior of the complex. Several studies support this notion: Gohlke and Klebe [29] emphasized the need to consider system flexibility and the biological environment. At the same time, Hou [30] noted that the accuracy of MMPBSA is highly dependent on simulation parameters such as duration. Additionally, Kollman [31] demonstrated that combining molecular dynamics with explicit solvation models significantly enhances the prediction of binding free energies and complex stability.

**Figure 5:**
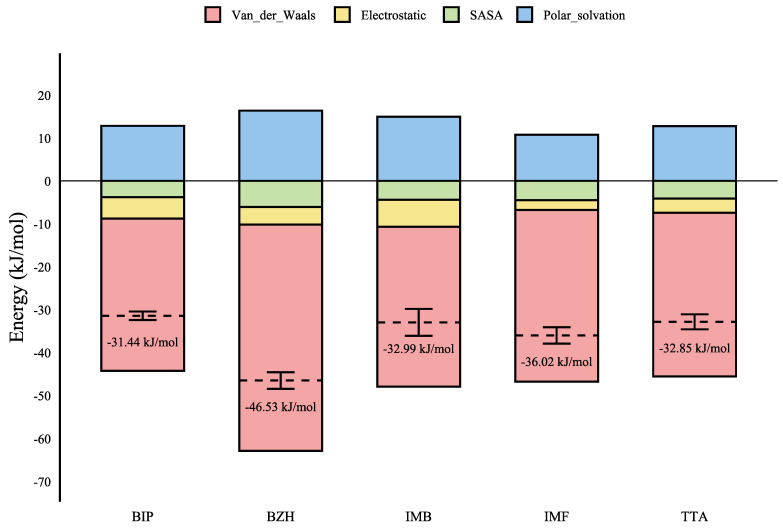
Energy decomposition of the binding energy of ligand-CK1δ complexes according to MMPBSA analysis.

In Figure 5, we presented the energetic decomposition of the ligand-CK1δ binding complexes, broken down into their main components: Van der Waals, electrostatics, polar solvation, and SASA for the BZH-CK1δ complex. It is important to emphasize that negative energies are contributive while positive energies are destabilizing. We observed that the interaction is primarily due to a substantial contribution from Van der Waals interactions, which represent the most significant component of the total binding energy.

**Figure 6:**
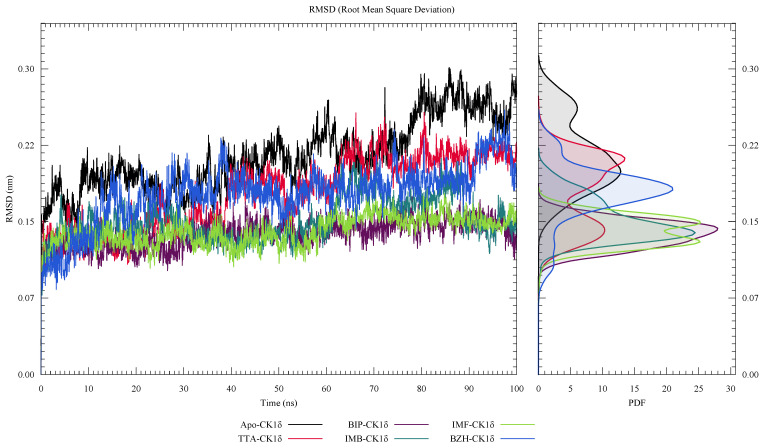
Root mean square deviation (RMSD) analysis of ligand-CK1δ complexes over a 100 ns molecular dynamics simulation.

In Figure 6, we showed the root mean square deviation (RMSD) analysis of ligand-CK1δ complexes during 100 ns of molecular dynamics simulation. All systems reach structural stability after the first 20 ns. BZH-CK1δ, BIP-CK1δ, and IMF-CK1δ complexes show more stable trajectories, whereas the IMB-CK1δ complex exhibits greater fluctuations over time. Furthermore, the probability density function (PDF) suggests that BZH-CK1δ, BIP-CK1δ, and IMF-CK1δ complexes present narrow and centered peaks, indicating structural stability. In contrast, IMB-CK1δ complexes show broad and dispersed distributions, reflecting higher conformational flexibility.

**Figure 7:**
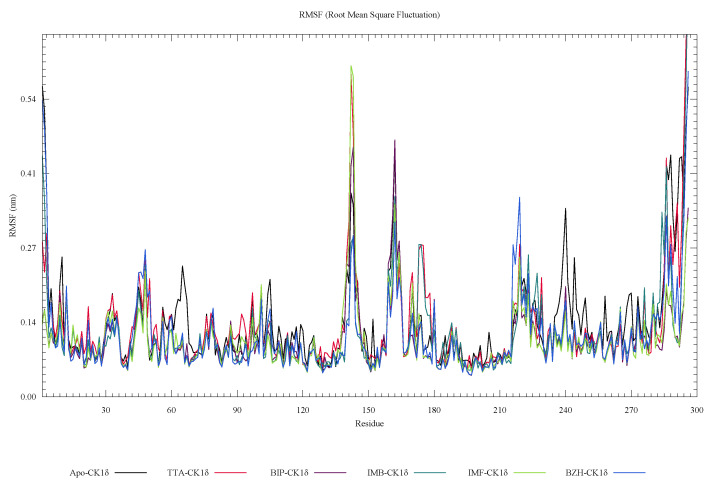
Residue mean square fluctuation (RMSF) analysis of ligand-CK1δ complexes during a 100 ns molecular dynamics simulation.

**Figure 8:**
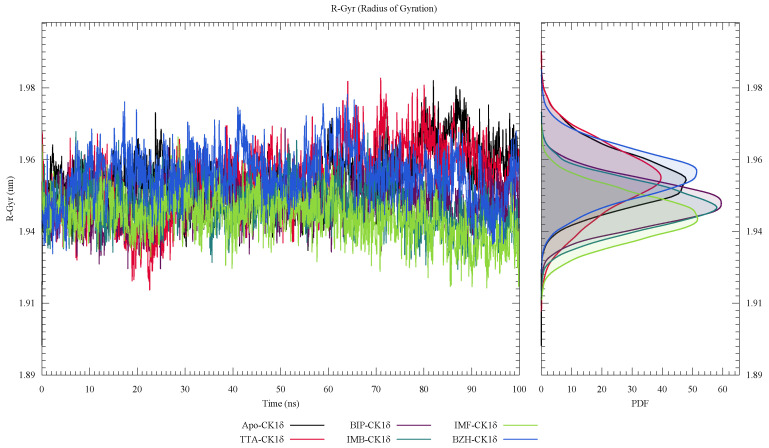
Radius of gyration (Rg) analysis of ligand-CK1δ complexes during a 100 ns molecular dynamics simulation.

In Figure 7, RMSF analysis shows that residual fluctuations are most pronounced in specific regions, around residues 140, 160, 220, and 240, suggesting localized flexibility that does not compromise the structural integrity of the system. Furthermore, in Figure 8, the radius of gyration (Rg) analysis shows that the IMF-CK1δ and BZH-CK1δ complexes present the lowest and most stable values over time, indicating greater structural compaction. In contrast, the TTA-CK1δ complex shows slightly higher values, suggesting less compaction. The PDF distribution reinforces these findings, with more concentrated peaks towards low values.

**Figure 9:**
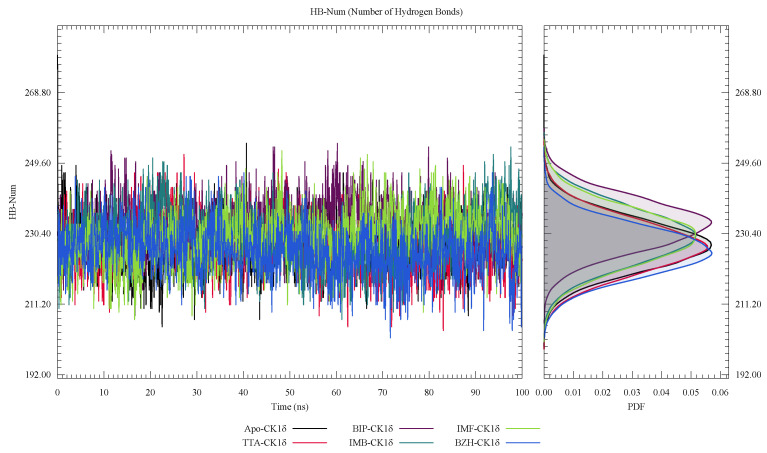
Number of hydrogen bonds (HBs) in ligand-CK1δ complexes during a 100 ns molecular dynamics simulation.

In Figure 9, we show that when analyzing the hydrogen bonds, they remain virtually unchanged throughout the trajectory, indicating that these interactions do not significantly influence the overall stability of the system during the simulation.

**Figure 10:**
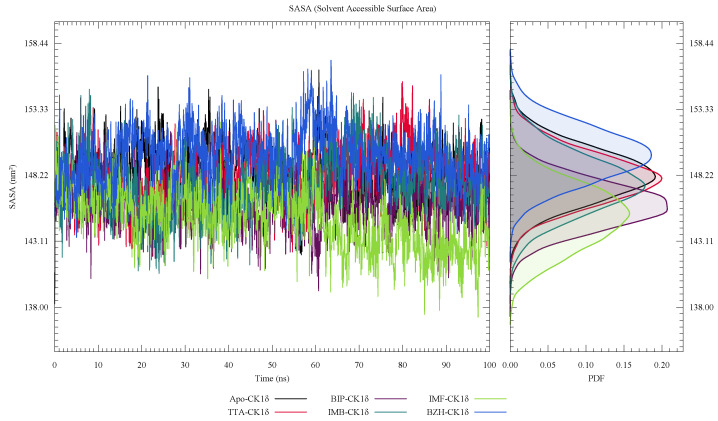
Solvent-accessible surface area (SASA) of ligand-CK1δ complexes during a 100 ns molecular dynamics simulation.

Similarly, in Figure 10, we observe that the SASA values remain relatively constant in all systems, without significant variations. It suggests that we are facing a general structural stability throughout the simulation.

**Figure 11:**
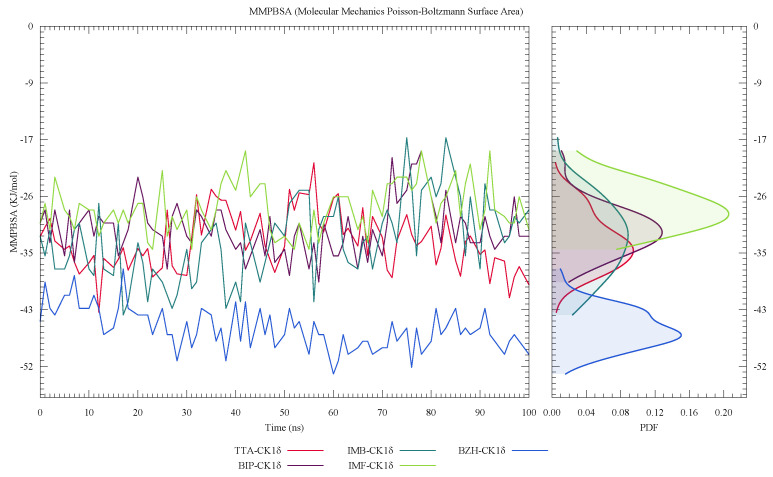
Binding free energy calculated by MMPBSA for ligand-CK1δ complexes during a 100 ns molecular dynamics simulation.

In Figure 11, we observed that the BZH-CK1δ complex maintains more negative and stable binding free energy values during the simulation, suggesting a stronger interaction with the protein. This result is consistent with the data presented in Table 2, where BZH presents the most favorable value, with −46.53 ± 1.94 kcal/mol. In contrast, the TTA-CK1δ, IMF-CK1δ, and IMB-CK1δ complexes present less negative values, and BIP-CK1δ has the highest, with −31.44 ± 0.99 kcal/mol, indicating a comparatively lower affinity. The PDF distribution confirms this trend, with a defined peak shifted towards low values for BZH.

**Figure 12:**
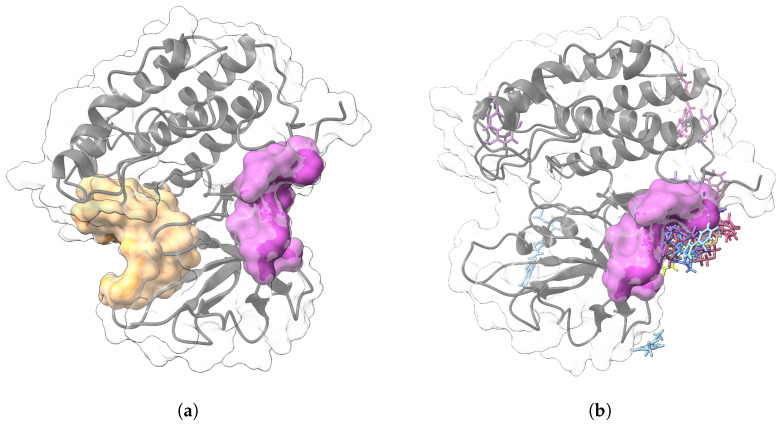
The best-scoring predicted protein pockets and ligand-preferred pockets in the protein after 100 ns of molecular dynamics simulation. (**a**) Representation of the pharmacological pockets predicted by CavityPlus, highlighting pocket 2 and pocket 6. (**b**) Final distribution of the ligands after 100 ns of molecular dynamics, demonstrating a common interaction zone with CK1δ.

In Figure 12a, we showed that pocket 2 was identified as the site with the highest druggable potential, while pocket 6 scored the lowest. However, Figure 12b shows that most ligands, following the molecular dynamics simulation, converge in the region corresponding to pocket 6. When analyzing the structural properties of CK1δ at the end of the molecular dynamics simulation, we considered only the five complexes with the better free energy of interaction.

**Figure 13:**
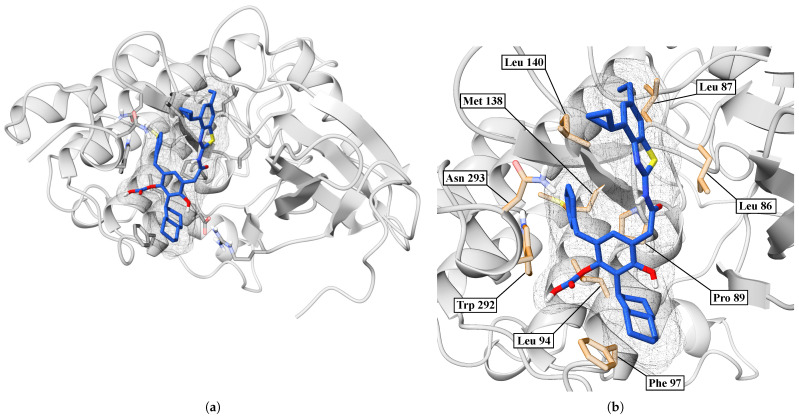
Interaction between CK1δ and the BZH ligand. (**a**) Overview. (**b**) Close-up.

In Figure 13a, we showed an overview of the BZH-CK1δ complex after 100 ns of molecular dynamics simulation, where we have found that the BZH ligand is embedded in a surface pocket of the protein. Figure 13b shows a close-up of the binding pocket, revealing specific interactions between BZH and several key residues. The ligand orientation allows for multiple hydrophobic and polar contacts.

**Figure 14:**
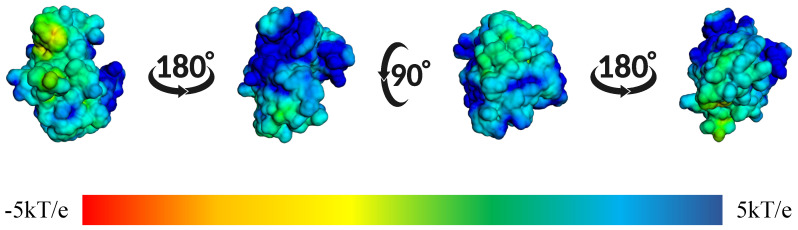
Mapping of the surface electrostatic potential of the BZH-CK1δ complex calculated using APBS after 100 ns of molecular dynamics. The different orientations show the potential distribution, where the blue regions represent positive charges and the red ones the negative charges, according to the kT/e scale.

In Figure 14, we observed the surface electrostatic potential mapping of the BZH-CK1δ complex after 100 ns of molecular dynamics. The binding site shows a predominantly blue and green distribution, indicating areas with positive or neutral charge, which means that these regions have positive or apolar amino acids.

**Figure 15:**
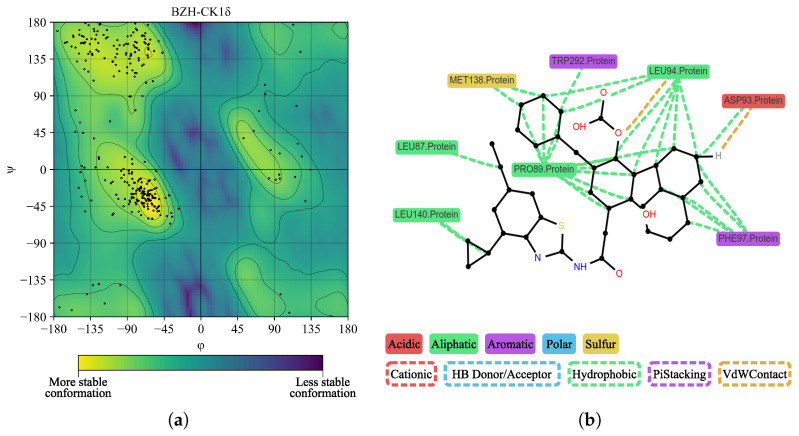
Ramachandran diagram and molecular interactions of the BZH-CK1δ complex after 100 ns of molecular dynamics. (**a**) Ramachandran diagram, the yellow color shows a more stable conformation and purple color shows a less stable conformation. (**b**) Molecular interactions.

In Figure 15a, we performed a Ramachandran plot analysis to assess the structural conformation of the BZH-CK1δ complex. Of the 261 non-glycine/proline residues analyzed, 218 residues (83.5%) were located in the most favored regions, 38 (14.6%) in additionally allowed regions, and 5 (1.9%) in generously allowed regions, representing 100% of the residues. We did not locate residues (0.0%) in the disallowed regions. This assessment excluded glycine and proline residues, as well as terminal residues, from the analysis. In Figure 15b, BZH interacts with several CK1δ residues, including LEU86, LEU87, PRO89, LEU94, PHE97, MET138, LEU140, TRP292, and ASN293. Although we did not identify hydrogen bonds, the average interaction distance was 3.7 Å.

**Figure 16:**
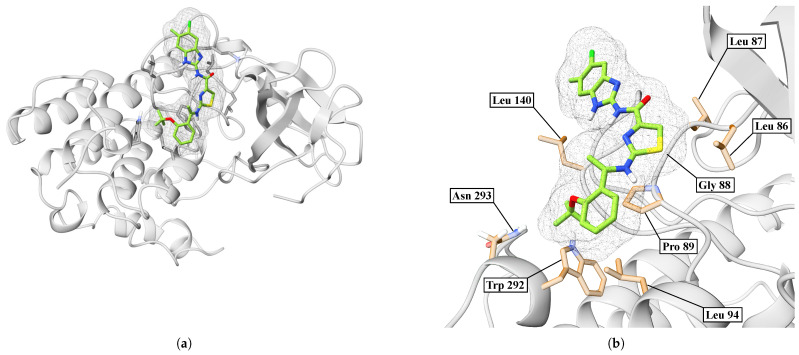
Interaction between CK1δ and the IMF ligand. (**a**) Overview. (**b**) Close-up.

In Figure 16a, we showed an overview of the IMF-CK1δ complex after 100 ns of molecular dynamics. In the case of the IMF, the ligand remains stably anchored in a cavity on the protein surface. On the other hand, Figure 16b shows a close-up of the binding site, where the IMF makes direct contact with key residues. The ligand orientation allows for multiple hydrophobic and polar contacts.

**Figure 17:**
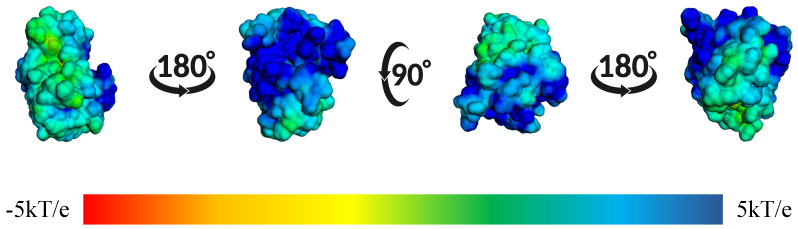
Mapping of the surface electrostatic potential of the IMF-CK1δ complex calculated using APBS after 100 ns of molecular dynamics. The different orientations show the potential distribution, where blue regions represent positive charges and red regions represent negative charges, according to the kT/e scale.

In Figure 17, we showed the surface electrostatic potential mapping of the IMF-CK1δ complex, calculated at the end of the molecular dynamics simulation. Rotated views of the protein reveal that the IMF binding site is surrounded primarily by regions of positive charge. The predominance of blue areas indicates positive electrostatic potentials, while the green-yellow areas correspond to neutral or slightly negative charges.

**Figure 18:**
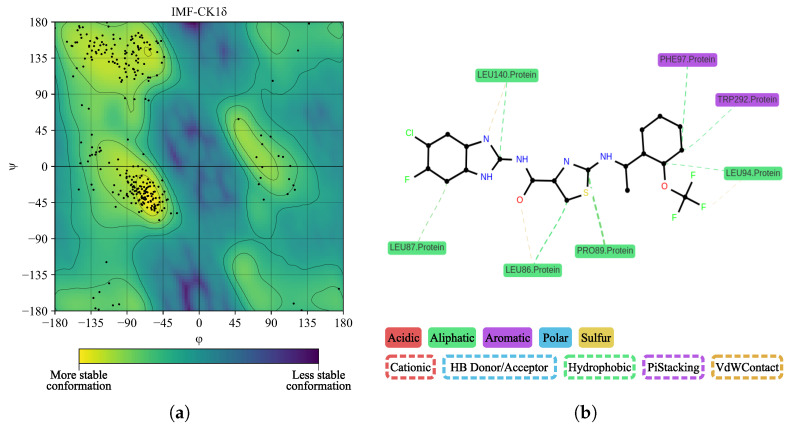
Ramachandran diagram and molecular interactions of the IMF-CK1δ complex after 100 ns of molecular dynamics simulations. (**a**) Ramachandran diagram, the yellow color shows a more stable conformation and purple color shows a less stable conformation. (**b**) Molecular interactions.

In Figure 18a, we performed a Ramachandran plot analysis to assess the structural conformation of the IMF-CK1δ complex. Of the 261 non-glycine/proline residues analyzed, 218 residues (83.5%) were located in the most favored regions, 39 (14.9%) in regions with extra permissions, and 3 (1.1%) in regions with generous permissions, representing 99.6% of the residues. We located only one residue (0.4%) in the regions with disallowed permissions. In Figure 18b, we observed interactions between BZH and several CK1δ residues, notably LEU86, LEU87, PRO89, LEU94, PHE97, MET138, LEU140, TRP292, and ASN293. We did not detect hydrogen bonds. The average interaction distance was 3.7 Å.

**Figure 19:**
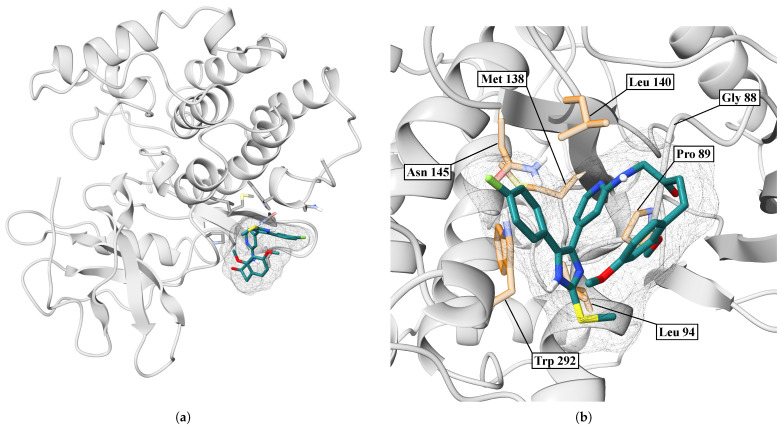
The IMB-CK1δ complex after 100 ns of molecular dynamics simulation, showing the key residues involved in the interaction with IMB. (**a**) Overview. (**b**) Close-up.

In Figure 19a, we showed a general view of the IMB-CK1δ complex after 100 ns of molecular dynamics simulation. The IMB ligand remains stably anchored in a cavity on the protein surface. Additionally, in Figure 19b, we showed a close-up of the binding site, where IMB enters direct contact with key residues.

**Figure 20:**
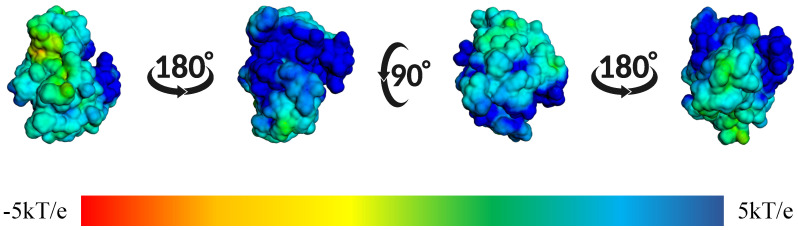
Mapping of the surface electrostatic potential of the IMB-CK1δ complex calculated using APBS after 100 ns of molecular dynamics. The different orientations show the potential distribution, where blue regions represent positive charges and red regions represent negative charges, according to the kT/e scale.

In addition, in Figure 20, we showed the surface electrostatic potential mapping of the IMB-CK1δ complex, calculated at the end of the molecular dynamics simulation. A clear predominance of blue areas is observed on the protein surface, suggesting a predominance of positive charges.

**Figure 21:**
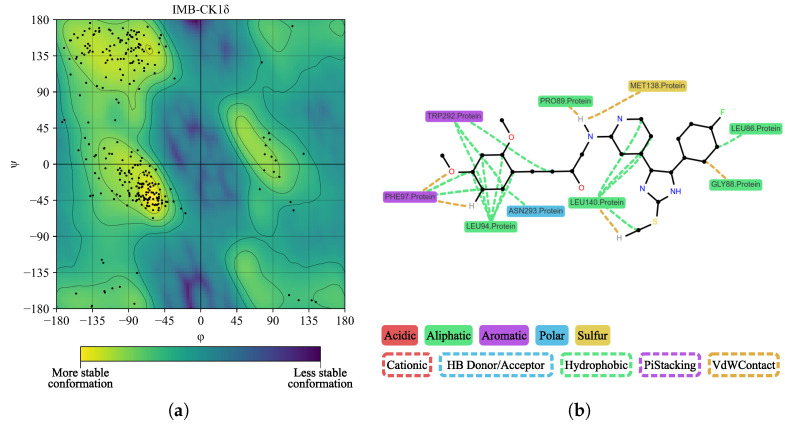
Ramachandran diagram and molecular interactions of the IMB-CK1δ complex after 100 ns of molecular dynamics simulation. (**a**) Ramachandran diagram, the yellow color shows a more stable conformation and purple color shows a less stable conformation. (**b**) Molecular interactions.

In Figure 21a, we performed a Ramachandran plot analysis to evaluate the structural conformation of the IMB-CK1δ complex. Of the 261 non-glycine/proline residues analyzed, 214 residues (82.0%) were located in the most favored regions, 40 (15.3%) in regions with extra permissions, and 5 (1.9%) in regions with generous permissions, representing 99.2% of the residues. We located only two residues (0.8%) in the disallowed regions. This evaluation excluded glycine and proline residues, as well as terminal residues, from the analysis. On the other hand, in Figure 21b, interactions between IMB and CK1δ residues are observed, including PRO89, LEU140, TRP292, MET138, GLY88, LEU94, and ASN145. We did not identify hydrogen bonds. The average contact distance was 3.64 Å.

**Figure 22:**
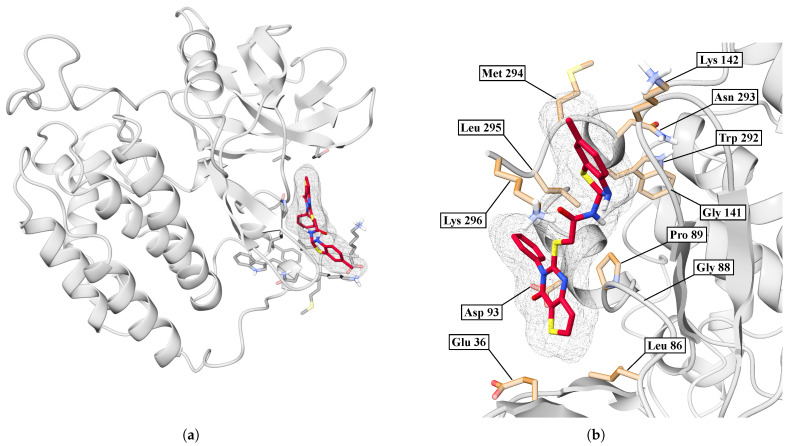
Interaction between CK1δ and the ligand TTA after 100 ns of molecular dynamics simulation. (**a**) Overview. (**b**) Close-up.

Likewise, in Figure 22a, we show an overview of the TTA-CK1δ complex after 100 ns of molecular dynamics simulation. The TTA ligand remains stably anchored in a cavity on the protein surface. Figure 22b shows a close-up of the binding site, where TTA makes direct contact with key residues.

**Figure 23:**
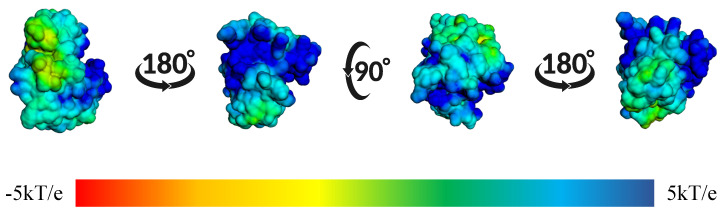
Mapping of the surface electrostatic potential of the TTA-CK1δ complex calculated using APBS after 100 ns of molecular dynamics. The different orientations show the potential distribution, where blue regions represent positive charges and red regions represent negative charges, according to the kT/e scale.

On the other hand, we showed in Figure 23 the surface electrostatic potential mapping of the TTA-CK1δ complex, calculated at the end of the molecular dynamics simulation. We can observe a clear predominance of blue areas on the protein surface, suggesting a predominance of positive charges or positive residues.

**Figure 24:**
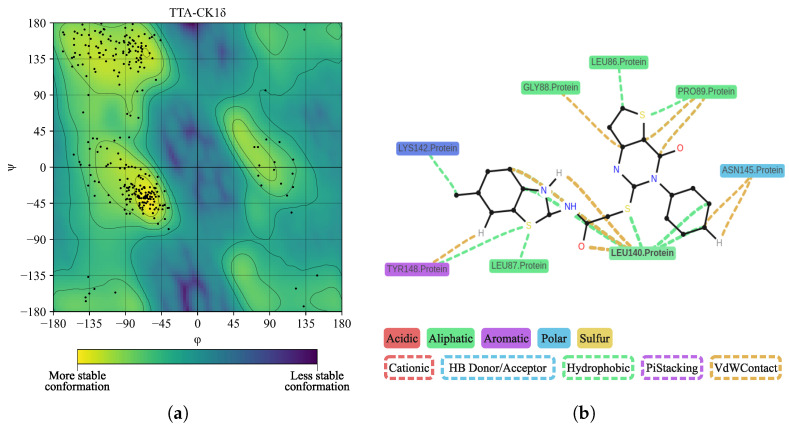
Ramachandran diagram and molecular interactions of the TTA-CK1δ complex after 100 ns of molecular dynamics simulation. (**a**) Ramachandran diagram, the yellow color shows a more stable conformation and purple color shows a less stable conformation. (**b**) Molecular interactions.

We performed a Ramachandran plot analysis, as shown in Figure 24a, to assess the structural conformation of the TTA-CK1δ complex. Of the 261 non-glycine/proline residues analyzed, 216 (82.8%) were located in the most favored regions, 40 (15.3%) in regions with additional permissions, and 5 (1.9%) in regions with generous permissions, representing 100% of the residues. We did not locate residues (0.0%) in the disallowed regions. This assessment excluded glycine and proline residues, as well as terminal residues, from the analysis. On the other hand, in Figure 24b, interactions between TTA and CK1δ residues, including LEU295, GLY88, LYS142, ASN293, GLU36, LEU86, PRO89, ASP93, GLY141, TRP292, MET294, and LYS296, are observed. We did not detect hydrogen bonds at this interface. The average contact distance was 3.67 Å.

**Figure 25:**
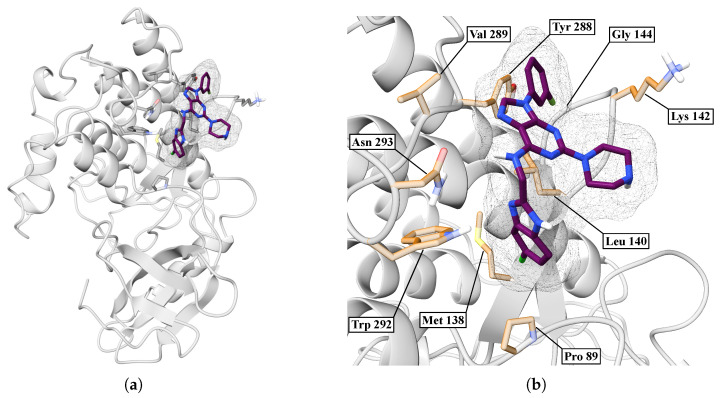
Interaction between CK1δ and the ligand BIP complex after 100 ns of molecular dynamics simulation. (**a**) Overview. (**b**) Close-up.

In Figure 25a, we showed an overview of the BIP-CK1δ complex after 100 ns of molecular dynamics simulation. The BIP ligand remains stably anchored in a cavity on the protein surface. Likewise, Figure 25b shows a close-up of the binding site, where BIP makes direct contact with key residues.

**Figure 26:**
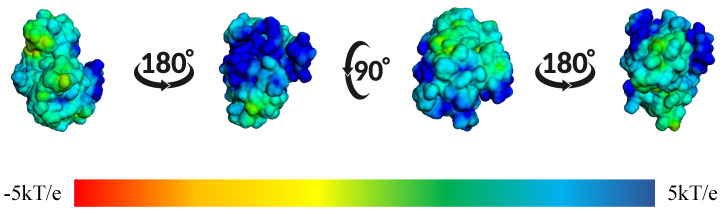
Mapping of the surface electrostatic potential of the BIP-CK1δ complex calculated using APBS after 100 ns of molecular dynamics. The different orientations show the potential distribution, where blue regions represent positive charges and red regions represent negative charges, according to the kT/e scale.

Additionally, we showed in Figure 26 the electrostatic surface potential of the BIP-CK1δ complex, calculated at the end of the molecular dynamics simulation. We observed the BIP ligand-binding pocket to be surrounded primarily by blue and green regions, indicating a predominantly positively charged zone with areas of neutral potential.

**Figure 27:**
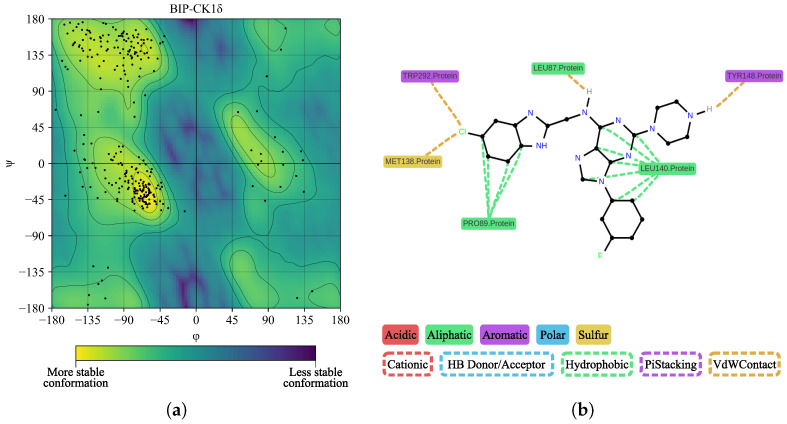
Ramachandran diagram and molecular interactions of the BIP-CK1δ complex after 100 ns of molecular dynamics simulation. (**a**) Ramachandran diagram, the yellow color shows a more stable conformation and purple color shows a less stable conformation. (**b**) Molecular interactions.

In Figure 27a, we performed a Ramachandran plot analysis to assess the structural conformation of the BIP-CK1δ complex. Of the 261 non-glycine/proline residues analyzed, 217 residues (83.1%) were located in the most favored regions, 37 (14.2%) in regions with extra permissions, and 6 (2.3%) in regions with generous permissions, representing 99.6% of the residues.

We located only one residue (0.4%) in the disallowed regions. This evaluation excluded glycine and proline residues, as well as terminal residues, from the analysis. In Figure 27b, interactions between BIP and CK1δ residues are observed, particularly TYR288, LEU140, GLY141, GLY144, PRO89, MET138, LYS142, TRP292, and ASN293. We detected only one hydrogen bond between BIP and residue GLY144. The average interaction distance was 3.65 Å.

Moreover, we found that BZH interacts with residues LEU86, LEU87, PRO89, LEU94, PHE97, MET138, LEU140, TRP292, and ASN293. According to Knippschild et al. [32], MET82 acts as a gatekeeper residue within the ATP-binding pocket of CK1δ. Also, Long et al. [33] identified LEU85, MET80, and MET82 as essential for inhibitor binding. In contrast, Córdova-Bahena et al. [34] and Sunkari et al. [35] confirmed the participation of GLU85 and LEU87 as relevant interaction sites. The presence of LEU87 among our interaction residues supports these findings (Table 3).

When we evaluated the pharmacokinetic properties (ADME) of BZH, IMF, IMB, TTA, and BIP using the ADMETlab 3.0 server [36]. All ligands, except IMB and BZH, complied with Lipinski’s rule, while none of them complied with Veber’s rule, which could affect their flexibility and oral bioavailability. Even so, all of them presented acceptable molecular weight values (MW < 650 Da), logP between 2.1 and 5.0, and TPSA < 110 Å^2^, which favored their absorption. Regarding the blood–brain barrier (BBB), IMB and BIP showed brain permeation capacity, while BZH, IMF, and TTA did not cross it (see Table 4).

**Figure 28:**
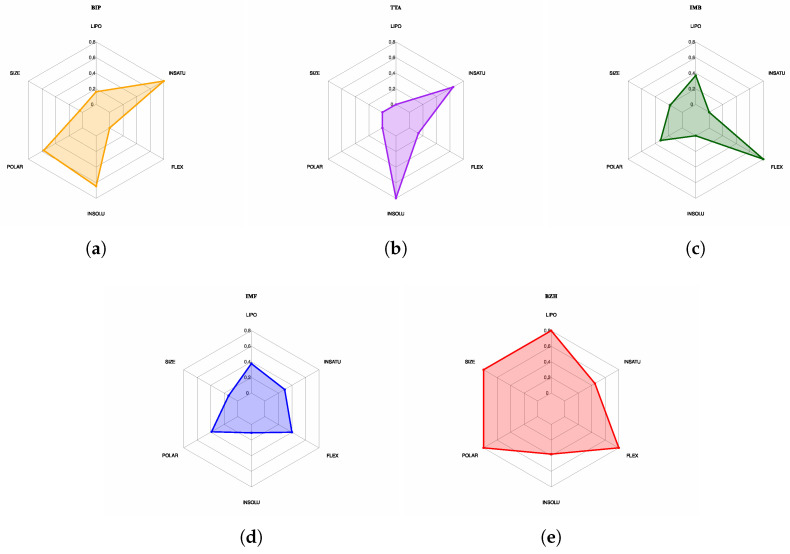
Oral bioavailability radar plots. The plots display six key properties: lipophilicity, size, polarity, solubility, flexibility, and saturation. (**a**) BIP. (**b**) TTA. (**c**) IMB. (**d**) IMF. (**e**) BZH.

We conduct a radar plot analysis, which reveals contrasting physicochemical profiles among the evaluated compounds. In Figure 28a, BIP displays high unsaturation (≈0.8) and moderate levels of polarity and insolubility, but low lipophilicity, size, and flexibility, suggesting a small and specific molecule, albeit with potential permeability limitations. In Figure 28b, TTA stands out for its high unsaturation (≈0.75) and insolubility (≈0.8), but with low lipophilicity, polarity, size, and flexibility values, indicating an unfavorable profile for oral bioavailability. In Figure 28c, IMB displays high flexibility (≈0.85) and moderate lipophilicity (≈0.55), accompanied by medium polarity and size, which may favor permeability, although its low unsaturation and insolubility limit its specificity. In Figure 28d, IMF shows a balanced profile with intermediate values in all properties, which positions it as one of the most promising candidates. In contrast, in Figure 28d, BZH exhibits extreme values in size, lipophilicity, polarity, and flexibility (all ≥ 0.9), with low insolubility (≈0.4), which configures an unfavorable profile for oral absorption. Finally, in Figure 28e, it is observed that IMF and BIP are the compounds with the most balanced profiles and suitable for bioavailability, while BZH, IMB, and TTA present properties that could compromise their pharmacological development.

**Figure 29:**
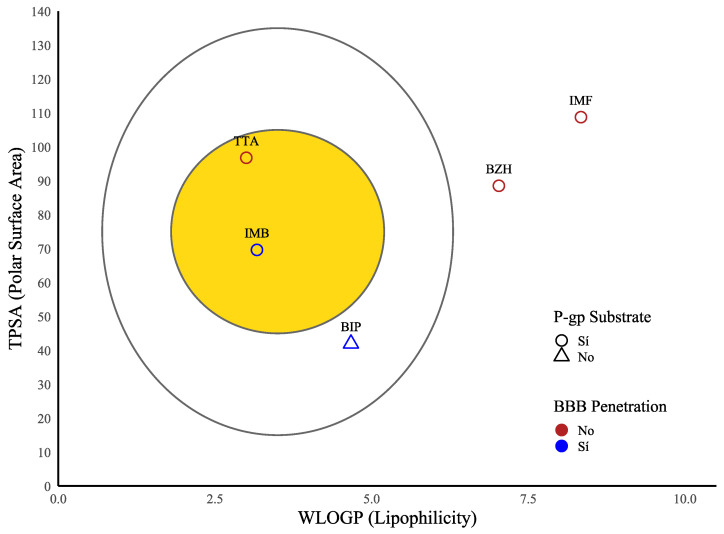
Boiled egg plot showing the lipophilicity (LogP) vs. the topological polar surface area (TPSA) of BIP, TTA, IMB, IMF, and BZH.

We evaluated bioavailability properties using the cooked egg model. We located BIP, IMF, IMB, and TTA in the yolk; however, given their characteristics, only BIP and IMF could cross the blood–brain barrier (BBB). In contrast, BZH, outside the egg, presents unfavorable physicochemical properties for both absorption and brain penetration. We classified BIP, IMF, and BZH as P-gp substrates, which favors their intracerebral permanence. From our analysis, we found that TTA and IMB compounds are actively excreted from the central nervous system (Figure 29).

Since safety is an essential requirement for drug development, the toxicity of BIP, TTA, IMB, IMF, and BZH compounds was assessed using the ADMETlab 3.0 platform [36]. According to the standard classification, all compounds were assigned to acute oral toxicity class IV, indicating moderate toxicity (LD50 between 300 and 2000 mg/kg). IMB and BZH presented the highest probabilities of hepatotoxicity (0.849 and 0.831, respectively), whereas all compounds showed low carcinogenicity (<0.06) and a moderate risk of neurotoxicity, with BIP being the highest in the latter aspect (0.271). Taken together, the results suggest that BIP and IMF possess a more favorable toxicological profile, whereas IMB and BZH might require further experimental validation to confirm their safety (Table 5).

From a pharmacokinetic perspective, IMF and BIP stand out as the most promising candidates, showing good oral absorption and the ability to cross the blood–brain barrier (BBB), as predicted by ADMETlab 3.0 [37]. Both comply with Lipinski’s rule [38] and exhibit a balanced physicochemical profile in terms of lipophilicity, size, polarity, and flexibility, as shown in Figure 28. In contrast, IMB and BZH do not meet key bioavailability criteria, showing properties that could limit their efficacy, such as low brain permeability or high molecular size. In terms of toxicity, all compounds were in class IV (LD_50_ between 300 and 2000 mg/kg), indicating a moderate risk. However, the IMF and BIP showed lower probabilities of causing liver or neurological damage. Overall, IMF emerges as the most balanced candidate for future applications in the central nervous system, followed by BIP.

We compared the experimental inhibitory activity (IC_50_) against CK1δ of the 26 compounds evaluated, which were compiled based on the available literature data. We summarized in Table 6 the binding free energy and IC_50_ values compounds, we observed an inverse relationship between binding free energy and IC_50_; with more favorable energies (≤−36 kcal/mol), such as BZH (−46.53 kcal/mol, IC_50_ ≈ 39.7 μM), IMF (−36.02 kcal/mol, IC_50_ = 0.085 μM), and IMB (−32.99 kcal/mol, IC_50_ = 0.004 μM), showed higher inhibitory potency. In contrast, molecules such as AMQ (−7.03 kcal/mol) and HYA (−19.63 kcal/mol) presented IC_50_ higher than 0.3 μM, suggesting a lower functional affinity. It is consistent with that reported by Walton et al. [39], who identified compounds capable of inhibiting CK1*δ* with very low IC_50_ concentrations.

Finally, it is essential to mention that, while static analysis with CavityPlus [71] identified pocket 2 as the most druggable (DrugScore = 1058), most ligands dynamically migrated towards pocket 6 during the simulation. We found that Bakan et al. [72] described this behavior by showing that structural flexibility allows some proteins to activate initially inactive regions. Furthermore, as reported by Córdova-Bahena et al. [34], docking can suggest an initial binding site, but only with molecular dynamics can it be confirmed whether the complex was truly stable and if that pocket was functional under dynamic conditions. These findings highlight the importance of complementing docking with molecular dynamics, since some regions only adopt a suitable shape to bind inhibitors when the protein is in motion. We presented results from the investigation that demonstrate the energetic efficiency of the CK1*δ*–ligand interaction and should not necessarily align with QSAR models as presented in ADMET.

## 3. Computational Methods

We downloaded the initial three-dimensional conformation of Cyclin-Dependent Kinase 1 delta (CK1δ) from the Protein Data Bank (PDB) (http://www.rcsb.org) [73,74], using the PDB ID: 6RCG [24]. We cleaned the molecule by removing all crystallographic water molecules. Missing fragments in the protein chain were reconstructed through homology modeling using MODELLER [26], integrated into the UCSF Chimera v1.18 interface [25]. The complete structure, free of artifacts and ready for subsequent simulations, was named “CK1δ”.

We selected the ligands studied through a literature review (Figure 3). Of the compounds selected for their occurrence in the literature regarding interaction with CK1δ, only one (AMI) is of natural origin; the rest are synthetic compounds in a preclinical research stage. For those whose structures were not available in databases such as PubChem [75] or DrugBank [76], we used the DECIMER server, a tool based on artificial intelligence for chemical structure recognition, to extract SMILES notation from graphical representations [77]. We verified the SMILES strings obtained and converted them into .sdf format using RDKit [78] in Python [79]; similar to the protein, we assigned each ligand a unique ID (Table 1). We performed Ligand parameterization and generation of molecular formats required for simulation locally using MglTools [80], AmberTools [81], and LigParGen [82], the latter applying the OPLS force field [83] to assign protein–ligand interaction parameters after docking.

Virtual screening of the ligands was performed on the known active site of the CK1δ protein using UNI-DOCK software, version 1.1 [28]. We configured the docking protocol to run 2500 iterations, generating 30 conformers per iteration for each ligand. We selected the conformers with the most favorable interaction energies for each ligand for further analysis.

We refined the atomic charges initially assigned by LigParGen [82] after docking for protein–ligand simulations. Subsequently, we calculated Hirshfeld charges using the CAM-B3LYP functional [84] in Gaussian 16 [85].

The structure of the CK1δ protein, as well as the protein–ligand complexes, was optimized and equilibrated through molecular dynamics simulations using GROMACS software, version 2024.5 [86,87]. We centered the protein system in a cubic geometry simulation box. We subsequently solvated the box with TIP3P water molecules [71]. To ensure the electrical neutrality of the system, chlorine (Cl^−^) and sodium (Na^+^) counterions were added. We initially minimized the system to resolve potential steric conflicts and reduce interatomic forces. Subsequently, we carried out two equilibrium stagest: a simulation in the NVT (constant number of particles, volume, temperature) ensemble at 309.65 K for 10 ns, followed by a simulation in the NPT (constant number of particles, pressure, temperature) ensemble at a pressure of 1 bar for 10 ns, ending with a molecular dynamics simulation (SDM) of 500 ns, we performed for the protein structure and the complexes the same procedure, varying only the SDM time to 100 ns for complexes.

Once we optimized the protein structure, we performed a preliminary analysis to identify and characterize potential ligand binding sites, focusing on pockets with a high probability of being pharmacologically relevant or interacting with ligands. This process was performed on the protein structure alone and post-interaction to verify possible changes in the pockets and interaction sites. We identified potential pockets and binding sites using CavityPlus [27].

The protein–ligand complexes obtained from the virtual screening were subjected to production molecular dynamics simulations using GROMACS software, version 2024.5 [86,87], following the optimization and equilibration steps described previously. We performed a 100 ns simulation in an isothermal-isobaric system.

We analyzed trajectories obtained from molecular dynamics simulations to assess the stability and dynamic behavior of the complexes. Metrics such as root mean square deviation (RMSD) [88], root mean square fluctuation (RMSF) [89], radius of gyration (Rg), and number of hydrogen bonds (HBs) were calculated, as well as the Solvent Accessible Surface Area (SASA) [86,86]. Additionally, we calculated the binding free energy of the complexes using the molecular mechanics Poisson–Boltzmann surface area (MMPBSA) method [90,91].

We identified specific interactions such as hydrogen bonds, Π–Π interactions, and hydrophobic contacts between ligands and key residues. We also assessed changes in surface electrostatic charge distribution using APBS [92] and ChimeraX visualization [37].

We predicted the pharmacokinetic properties and ADMET parameters of the candidate ligands using ADMETlab 3.0 [36]. We analyzed key descriptors such as molecular weight, logP, TPSA, HBD, HBA, and rotatable bonds to assess drug-likeness based on Lipinski’s and Veber’s rules. The boiled egg model was used to predict gastrointestinal absorption, blood–brain barrier permeability, and P-glycoprotein interactions. Additionally, in silico toxicity assessments included hepatotoxicity, neurotoxicity, carcinogenicity, and acute oral toxicity (LD_50_).

We assessed the stereochemical quality of the structures using Ramachandran diagrams generated with Ramachandraw [93]. We calculated electrostatic potentials with APBS [92]. We performed 3D visualization and structural manipulation with ChimeraX [37] and RDKit [78]. We used Open Babel for chemical structure processing, and we plotted numerical results with Gnuplot [94].

## 4. Conclusions

In this study, we identified BZH as the most stable inhibitor of the CK1δ protein, following a combined docking, molecular dynamics, and free energy analysis (MMPBSA), highlighting its interaction with key catalytic residues. We found that the compounds with the best binding typically have lower IC_50_, confirming the usefulness of the computational approach in predicting efficacy. IMF and BIP stand out as the most promising candidates due to their good absorption and ability to reach the brain, unlike others, such as IMB, which, although potent, may not be effective in the body. Finally, we demonstrated that molecular dynamics simulation allows the identification of functional regions not visible in static analyses, such as pocket 6, highlighting the importance of complementing docking with dynamic simulations for a more realistic and accurate prediction of ligand-protein behavior.

## Figures and Tables

**Table 1:** Analysis of druggable pockets of the CK1*δ* protein.

Protein	Pocket	DrugScore	Druggability
CK1*δ*	2	1058	Strong
	4	22	Medium
	1	14	Medium
	5	−375	Weak
	3	−651	Weak
	7	−782	Weak
	8	−1200	Weak
	9	−1183	Weak
	6	−1274	Weak

**Table 2:** Docking and MMPBSA energy components for ligand binding to CK1δ (kcal/mol).

Ligand	Dock	MMPBSA Components					
		VdW	Elec	Pol	SASA	SAV	Total
BZH	−7.663	−52.70±1.93	−4.12±0.12	16.37±0.05	−6.07±0.09	10.30±0.10	−46.53±1.94
IMF	−7.171	−40.00±1.36	−2.27±1.28	10.76±0.45	−4.51±0.01	6.25±0.45	−36.02±1.92
IMB	−6.806	−37.25±3.07	−6.30±0.62	14.96±0.15	−4.41±0.21	10.56±0.26	−32.99±3.14
TTA	−6.533	−38.15±1.03	−3.32±1.29	12.76±0.58	−4.13±0.02	8.62±0.58	−32.85±1.75
BIP	−7.322	−35.48±0.94	−5.00±0.18	12.83±0.24	−3.79±0.01	9.04±0.24	−31.44±0.99
BZC	−7.076	−36.14±0.79	−0.61±0.08	9.80±0.20	−4.23±0.10	5.57±0.22	−31.18±0.83
IMT	−7.211	−34.69±1.59	1.65±1.36	6.49±1.02	−3.87±0.04	2.62±1.02	−30.43±2.33
IMD	−7.035	−33.19±0.73	−9.09±0.18	15.88±0.08	−3.71±0.06	12.17±0.10	−30.11±0.75
BIM	−7.704	−34.46±1.43	−1.85±0.32	10.61±0.37	−3.93±0.10	6.68±0.38	−29.63±1.51
PMP	−6.103	−31.87±0.42	−4.68±0.20	11.32±0.30	−3.66±0.02	7.66±0.30	−28.89±0.55
PYP	−7.104	−32.66±0.71	0.80±0.23	7.42±0.06	−4.17±0.03	3.26±0.07	−28.61±0.75
LAX	−6.517	−32.81±1.64	−2.43±0.64	10.24±1.25	−3.51±0.06	6.73±1.26	−28.51±2.16
LAM	−6.813	−32.58±1.25	2.32±0.97	5.26±0.38	−3.27±0.00	1.99±0.38	−28.27±1.63
BIT	−6.685	−32.82±1.38	−3.60±0.05	11.81±0.37	−3.55±0.04	8.26±0.37	−28.16±1.43
BIC	−6.090	−30.74±0.91	−1.40±0.33	8.79±0.68	−3.85±0.00	4.94±0.68	−27.19±1.19
PYR	−6.093	−28.74±0.38	−1.37±0.16	6.39±0.09	−3.23±0.07	3.16±0.11	−26.95±0.42
PYI	−6.885	−27.40±0.90	−0.29±0.03	6.74±0.02	−3.48±0.06	3.26±0.06	−24.42±0.90
AMI	−5.570	−27.42±0.23	−4.38±1.38	10.75±0.63	−3.16±0.15	7.58±0.65	−24.22±1.54
BZF	−6.044	−27.48±0.59	−3.83±0.88	11.05±0.75	−3.60±0.03	7.45±0.75	−23.86±1.30
BZT	−6.090	−26.35±0.32	−1.20±0.77	7.09±0.49	−3.34±0.02	3.75±0.49	−23.79±0.97
BZM	−6.004	−26.11±0.36	−1.42±0.81	7.34±0.58	−3.00±0.01	4.34±0.58	−23.19±1.06
DXB	−8.137	−26.02±0.77	−11.40±2.75	20.33±1.93	−3.07±0.04	17.25±1.93	−20.16±3.45
HYA	−5.648	−21.52±0.43	−2.90±0.37	7.31±0.43	−2.52±0.01	4.79±0.43	−19.63±0.71
AHQ	−5.276	−23.37±1.28	−2.04±0.58	9.52±0.10	−2.69±0.08	6.83±0.13	−18.57±1.41
DXM	−6.435	−17.90±0.63	−1.61±1.91	6.70±1.71	−2.28±0.00	4.42±1.71	−15.09±2.64
AMQ	−5.273	−7.85±0.31	−0.52±0.58	2.32±0.19	−0.97±0.21	1.35±0.28	−7.03±0.71

**Dock**: Docking score. **VdW**: Van der Waals energy. **Elec**: Electrostatic energy. **Pol**: Polar solvation energy. **SASA**: Solvent-accessible surface area. **SAV**: Solvation energy. **Total**: Total MMPBSA energy. All values in kcal/mol; errors are ± standard deviation.

**Table 3:** Summary of the interactions between BZH, IMF, IMB, TTA, and BIP with the amino acid residues within the active site of CK1δ.

Ligand	Interacting Residues	H. Bonds	Dist. (Å)
BZH	LEU86, LEU87, PRO89, LEU94, PHE97, MET138, LEU140, TRP292, ASN293	—	3.70
IMF	LEU86, LEU87, GLY88, PRO89, LEU94, LEU140, TRP292, ASN293	—	3.64
IMB	PRO89, LEU140, TRP292, MET138, GLY88, LEU94, ASN145	—	3.64
TTA	LEU295, GLY88, LYS142, ASN293, GLU36, LEU86, PRO89, ASP93, GLY141, TRP292, MET294, LYS296	—	3.67
BIP	TYR288, LEU140, GLY141, GLY144, PRO89, MET138, LYS142, TRP292, ASN293	GLY144	3.65

**Table 4:** Pharmacokinetic parameter evaluation (ADME) of CK1δ ligands.

Ligand	MW	HBD	HBA	LogP	TPSA	RB	Lipinski	Veber	HIA	BBB
BIP	477.16	3	9	3.384	99.58	5	Accepted	—	—	+++
TTA	468.07	2	6	2.113	76.02	6	Accepted	—	—	—
IMB	506.18	2	7	5.006	89.13	11	Rejected	—	—	—
IMF	497.03	3	7	5.054	91.93	8	Accepted	—	—	++
BZH	638.28	3	7	8.339	108.75	11	Rejected	—	—	—

MW: molecular weight in Da; HBD: hydrogen bond donors; HBA: hydrogen bond acceptors; TPSA: topological polar surface area; RB: rotatable bonds; HIA: human intestinal absorption; BBB: blood–brain barrier permeability.

**Table 5:** Toxicity assessment results of BIP, TTA, IMB, IMF, and BZH analyzed using ADMETlab 3.0.

Compound	Hepatotoxicity	Carcinogenicity	Neurotoxicity	LD_50_ (Oral, Rat, mol/kg)	Toxicity Class
BIP	0.768	0.040	0.271	0.503	IV
TTA	0.715	0.052	0.251	0.550	IV
IMB	0.849	0.032	0.212	0.497	IV
IMF	0.812	0.037	0.242	0.512	IV
BZH	0.831	0.039	0.262	0.567	IV

All toxicity predictions are based on in silico models. LD_50_ values are given in mol/kg. Toxicity classes follow the globally harmonized system (GHS).

**Table 6:** Binding free energies and IC_50_ values of selected compounds against CK1δ.

Compound	Binding Energy (kcal/mol)	IC_50_ (μM)	Reference
BZH	−46.53±1.94	≈39.7000	[40]
IMF	−36.02±1.92	0.0850	[41]
IMB	−32.99±3.14	0.0040	[42]
TTA	−32.85±1.75	0.9300	[43]
BIP	−31.44±0.99	0.0040	[44]
BZC	−31.18±0.83	≈34.0000	[40]
IMT	−30.43±2.33	0.0029	[41,45]
IMD	−30.11±0.75	0.3000	[44,45,46]
BIM	−29.63±1.51	0.0440	[44,47]
PMP	−28.89±0.55	0.0005–0.0200	[48]
PYP	−28.61±0.75	0.0039	[49,50]
LAX	−28.51±2.16	0.8000	[51]
LAM	−28.27±1.63	0.4100	[51]
BIT	−28.16±1.43	0.0440	[52]
BIC	−27.19±1.19	2.3200	[53]
PYR	−26.95±0.42	0.7000	[39,54]
PYI	−24.42±0.90	0.0130	[39,44,55,56,57,58]
AMI	−24.22±1.54	0.7000	[59]
BZF	−23.86±1.30	47.0000	[60,61,62]
BZT	−23.79±0.97	0.0230	[22,60,61,63]
BZM	−23.19±1.06	0.2900	[64]
DXB	−20.16±3.45	0.0200	[65]
HYA	−19.63±0.71	0.0300	[66,67,68,69]
AHQ	−18.57±1.41	0.6600	[70]
DXM	−15.09±2.64	0.0700	[65]
AMQ	−7.03±0.71	0.3300	[64]

Binding energies were computed from molecular dynamics simulation. IC_50_ values were obtained from experimental sources.

## Data Availability

The original contributions presented in this study are included in the article. Further inquiries can be directed to the corresponding author.
